# Single-mode interface states in heterostructure waveguides with Bragg and non-Bragg gaps

**DOI:** 10.1038/srep44381

**Published:** 2017-03-13

**Authors:** Ya-Xian Fan, Tang-Qing Sang, Ting Liu, Lan-Lan Xu, Zhi-Yong Tao

**Affiliations:** 1 Key Lab of In-fiber Integrated Optics, Ministry Education of China, Harbin Engineering University, Harbin 150001, People’s Republic of China; 2 Photonics Research Centre, College of Science, Harbin Engineering University, Harbin 150001, People’s Republic of China; 3 Physics Research Centre, College of Science, Harbin Engineering University, Harbin 150001, People’s Republic of China

## Abstract

Interface states can always arise in heterostructures that consist of two or more (artificial) materials with topologically different energy bands. The gapped band structure can be classified by the Chern number (a topological invariant) generally or the Zak phase in one-dimensional periodic systems. Recently, topological properties have been employed to investigate the interface states occurring at the connecting regions of the heterostructures of mechanical isostatic lattices and acoustical waveguides. Here, we study this heterostructure phenomenon by carefully connecting two corrugated stainless steel waveguides with Bragg and non-Bragg gaps at approximately the same frequency. These two waveguide structures can be achieved by continuously varying their geometry parameters when a topological transition exists in the forbidden bands, in which the reflection impedance changes the sign. Furthermore, a localized single high-order mode has been observed at the interface because of the transverse mode interactions, which relate to the non-Bragg gaps created by the different transverse mode resonances. Such a localized acoustic single mode with very large enhanced intensity could find its applications in sound detection, biomedical imaging, and underwater sound control, and could also enrich our means of wave front manipulations in various engineering fields.

Heterostructures, the combinations of multiple semiconductor materials with unequal band gaps^1^, have been developed to indicate any combined materials or structures with energy or frequency gaps^2^,^3^,^4^,^5^,^6^,^7^. Between each layer or region of heterostructures, the interface states can always arise to localize the energy, which has been known as the Tamm states in solid state physics^8^. In photonic crystal heterostructures, the interface states were investigated numerically^9^ and experimentally^10^ and provided the very high performance nanostructure devices^11^,^12^. The existence of gapless states at interfaces, related to topological insulators^13^,^14^,^15^, quantum Hall effect^16^,^17^, Tamm plasmon- polaritons^18^,^19^ etc., can be attributed to the topological classification of gaps, whose topological invariants are different.

The topological classification provides the equivalence states when the Bloch Hamiltonian changes with varying geometry parameters, and the bulk band structures can be identified by the geometry phase (Berry’s phase) or the Chern invariant, in terms of holonomy^13^,^20^. In one-dimensional cases, Berry’s phase has been investigated and simplified as the Zak phase^21^,^22^ using the Wannier functions^23^. In 2014, Kane & Lubensky introduced the topological notations to the mechanical isostatic lattices and investigated the topological protection in one- and two- dimensional model systems^24^. In 2015, Xiao *et al*. demonstrated the Zak phase concept in a combined acoustic waveguide system^25^. In quantum and classical physics, connecting several structures (or materials) with topological different band gaps has led to so many interface states with exciting physical concepts and functional materials. However, the transverse modes and their interactions at interface states have not been investigated. When the waveguide heterostructures are elaborated, the transverse modes cannot be ignored any more at their interfaces. The transverse mode resonances and related gaps are always very complex and intriguing in a waveguide with periodically undulated walls. The Bragg gaps are created by the same transverse mode resonances, while the coupling of different transverse modes will result in the so-called non-Bragg gaps, which have been found and investigated theoretically^26^ and experimentally^27^,^28^. The investigations on the topological properties of the Bragg and non-Bragg gaps are essential and interesting to enrich our knowledge on the topology and its applications.

Here, we simply connect two different acoustic waveguides to demonstrate the creation of the acoustical heterostructures and the related interface states. When the geometry parameters of a waveguide vary, the topological characteristics of the Bragg and non-Bragg gaps are invariant but their frequency ranges would shift. Thus, we can select the same frequency for the Bragg and non-Bragg gaps and elaborate the acoustic heterostructure waveguides by carefully connecting two structures. Because of the different topologies, the interface states will arise with the enhanced sound intensity in the former forbidden bands. What is more intriguing is that the localized interface states can consist of a single high-order mode owing to mode coupling in non-Bragg gaps, which will be demonstrated numerically and experimentally in the following. The localized single mode could find its applications in various fields, such as trap and control of microparticals, boundary control of soft matter, and imaging.

## Results

### Topological differences of Bragg and non-Bragg gaps

**Figure 1:**
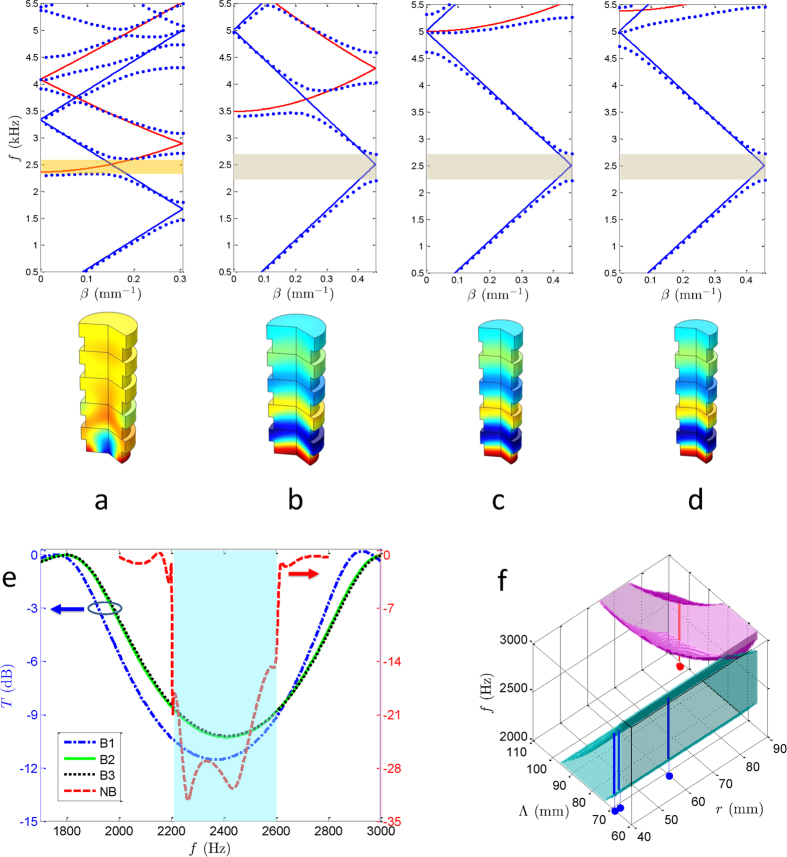
Band alignment of the Bragg and non-Bragg gaps with different topological characteristics. (**a–d**) Characteristic dispersion curves (top) and simulated sound pressures at 2349 Hz (bottom). The blue and red solid lines denote the fundamental and first modes, respectively, whereas the blue bold dots depict the dispersion curves of four different waveguides. (**a**) NB with non-Bragg gap at approximately 2500 Hz, shown by the yellow shadow. (**b–d**) Bragg waveguides with Bragg gap at approximately 2500 Hz, shown by the grey shadows. The Bragg waveguides with slight difference can be identified by the cutoffs of the first mode (red lines). (**e**) Transmission coefficients of the non-Bragg and three Bragg waveguides, denoted by the red dashed, blue dash-dot, black dotted, and green solid lines, respectively. It is clear that the Bragg gaps are much wider than the non-Bragg one but less efficient in sound attenuation. In any case, a common gap exists for the four waveguides in the frequency range of 2200 to 2600 Hz, denoted by the light blue shadow. (**f**) Band gaps between 2000 and 3000 Hz vs. different geometrical parameters (the mean radius and period of the waveguides). The magenta and cyan volumes denote the different topological characteristics of the non-Bragg and Bragg gaps, respectively. The bold dots present the geometric parameters of the waveguides investigated, whereas the bold lines denote the related band widths.

The band alignment for the four different waveguides is shown in Fig. 1a–d. The solid lines depict the characteristic lines for the corrugated waveguide^26^ with a mean radius *r*_0_ and period Λ and


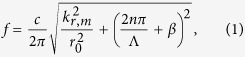


where *c* is the speed of sound, *β* is the propagation constant, and the transverse wavenumber *k*_*r,m*_ is a zero of the first-order Bessel function. The blue solid lines denote the fundamental modes for integer *n* with *k*_*r*,0_ = 0, whereas the red solid lines show the first mode with *k*_*r*,1_ = 3.8317. It has been determined that the resonances will occur at the intersections of these characteristic lines and result in the band gaps. The same mode intersections lead to Bragg resonances, whereas different ones result in non-Bragg resonances^21^. Setting the resonant frequency to 2500 Hz and considering the non-Bragg resonance in Fig. 1a yields the non-Bragg waveguide (NB) with geometric parameters of Λ = 102.9 mm and *r*_0_ = 88.76 mm. At the same resonant frequency, we obtain the three Bragg waveguides (B1-B3) with different cutoffs of the first mode. The wall periods of the three waveguides are selected as Λ = 68.6 mm; *r*_0_ = 60 mm for B1, *r*_0_ = 41.85 mm for B2, and *r*_0_ = 40 mm for B3. Selecting the 10% corrugation of the mean radius, we calculated the dispersion curves and depict them in the upper sections of Fig. 1a–d using bold blue dots. The shadows between the dispersion curves indicate the non-Bragg and Bragg gaps at approximately 2500 Hz.

Using the finite element method (FEM) with COMSOL Multiphysics, we have calculated the sound pressure in the band gap (2349 Hz), as presented in the bottom sections of Fig. 1a–d, showing the differences between the non-Bragg and Bragg gaps, i.e., that the transverse variations are induced in the non-Bragg gap. The transmission coefficient is


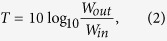


where the inlet and outlet sound power are denoted by *W*_*in*_ and *W*_*out*_, respectively. It can be observed in Fig. 1e that the Bragg gaps are much wider than the non-Bragg one but less efficient with respect to sound attenuation. The light blue shadow indicates a common gap for the four waveguides in the frequency range from 2200 to 2600 Hz. At these frequencies, a sound wave cannot propagate through any of these four waveguides.

In addition to the band width and attenuation efficiency, which can be found easily in the transmission coefficient calculations, the topological analysis of these different waveguides can provide more intrinsic characteristics of the Bragg and non-Bragg gaps. Using the Wannier functions, the topological classification can be identified by the Zak phase^21^, which reveals the sign of the relative reflection impedance^29^; i.e.,


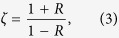


where *R* is the reflection coefficient of the waveguide at its ends. Positive or negative impedance indicates different topological properties. We have investigated the sign of the impedance for the Bragg and non-Bragg gaps and calculated the gap edges via the perturbation method (see the Supplementary Material). The band gaps and their topological identifications for frequencies between 2000 and 3000 Hz are depicted in Fig. 1f. The magenta and cyan volumes denote the different topological characteristics of the non-Bragg and Bragg gaps, respectively. When the geometric parameters vary, the Bragg and non-Bragg gaps change in the cyan and magenta volumes, respectively. However, the topological properties are invariant, displaying the identical colour in each volume.

### Acoustical interface states and their mode characteristics

**Figure 2:**
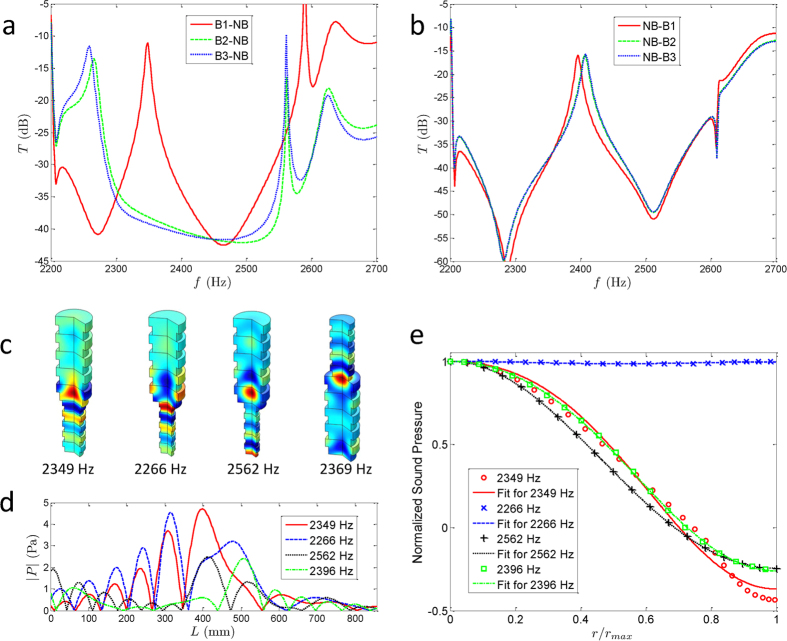
Interface states and their mode characteristics. (**a**) Transmission coefficients of heterostructure waveguides, with the NB connected after the Bragg ones. Two peaks appear in the former band gap for each connection. Only one peak at 2349 Hz for the connection of B1-NB is in the centre of the band gap; the others are all near the band edges. (**b**) Transmission coefficients of heterostructure waveguides, with the Bragg waveguides connected after the non-Bragg one. One peak appears in the centre of the gap for each connection, but the transmission coefficients are all smaller than −15 dB. (**c**) Sound pressure for the heterostructure waveguides, with B1-NB at 2349 Hz, B2-NB at 2266 Hz, B3-NB at 2562 Hz, and NB-B1 at 2396 Hz. The maximum magnitudes of the interface states appear at different locations with substantially different mode structures for the four heterostructure waveguides. (**d**) Absolute value of sound pressure along the symmetry axis of the heterostructure waveguides. The red solid, blue dashed, black dotted, and green dash-dot lines represent the four heterostructure waveguides in **c**, respectively. (**e**) Normalized sound pressure with its best fit along the radius at the location of the interface states. The mode structures can be identified by the mean square fit method; the single fundamental and first modes can be achieved in heterostructure B2-NB and others.

Because of the different topological characteristics of the Bragg and non-Bragg gaps, we have connected the Bragg and non-Bragg waveguides in two different ways to form the heterostructures. When we constructed the heterostructures, we had to keep the sound propagation direction in each waveguide unchanged as in Fig. 1, which guarantees the frequency of interface state falls in the former gap. At this frequency, the relative reflection impedance of two topological different waveguides is the additive inverse of each other^29^. In other way of heterostructure formation, the gapless interface state shifts too close to the band edge to be figured out distinctly. For the Bragg waveguide positioned first, the calculated transmission coefficient of the heterostructure waveguide is shown in Fig. 2a. Two peaks appear in the former band gap for each structure. In these peaks, only one at 2349 Hz for the heterostructure of B1-NB is in the centre of the gap; the others are all near the band edges. When the NB is positioned first, the transmission coefficients exhibit substantially different properties: only one peak appears in the former gap in Fig. 2b instead of two. Although the interface states are induced by the connection of two waveguides with different topological characteristics, the transmission coefficients are all smaller than −15 dB.

Using FEM, the sound pressures at the frequency of interface states have been simulated (see the Supplementary Material); some of them are presented in Fig. 2c. It can be clearly observed that the maximum amplitudes of sound pressures appear at different locations with considerably different mode structures for the four heterostructure waveguides. Because the high-order mode creates non-Bragg gaps, the interface states exhibit not only the accumulation of the sound energy but also the different mode structures comparing with Fig. 1. The absolute values of sound pressure along the symmetry axis of the heterostructure waveguides, shown in Fig. 2d, illustrate the sound energy accumulation at the interface with very large enhanced intensity beyond the incident one.

To further investigate the mode structure of the interface states, we choose the longitudinal location of the maximum pressure, determine the sound pressure along the radius direction, and analyse its components. The sound pressures, normalized by their own maximum along the radius, are depicted in Fig. 2e. The heterostructure of B2-NB exhibits nearly unit radius distribution, inhabiting only the interface state that consists of the fundamental mode, whereas the other three structures can provide higher mode interface states. To identify the mode structure of the interface states, we performed a least-square fit to the normalized sound pressures with the model, i.e.,


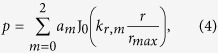


where J_0_(·) is the zeroth-order Bessel function and the coefficient *a*_*m*_, describing the relative content of the different modes, satisfies 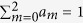 because of the normalization. The best fits along the radius for the sound pressures are also depicted in Fig. 2e; the related optimal coefficients are given in Table 1. The mode structures can be identified by the values of optimal coefficients; the single fundamental and first modes have been achieved, respectively, in the heterostructure of B2-NB and others. In the non-Bragg gaps, both the fundamental and first modes resonate and attenuate, whereas only the fundamental modes are involved in the Bragg gaps. Additionally, the first mode cannot propagate from the NB to the B1, B2, or B3 because of its higher cutoff in the Bragg waveguides. Thus, the transverse mode resonances result in the localized single first mode at the interfaces. But there is an exception that the localized fundamental mode arises at 2266 Hz in the heterostructure of B2-NB. It should be noted that the interface state appears in the B2 and the frequency is much smaller than the cutoff (2357 Hz) of the first mode in the NB, which means that the first mode is not strong enough to survive in such interface state. Except for the heterostructure of B2-NB, we have obtained an interface state at the connecting area of the two periodic acoustic waveguides along with a single high-order transverse mode with very large enhanced intensity at the interface. These investigations of the interface states induced by connecting two periodic structures with band gaps displaying different topological properties reveal the mechanisms of mode interactions and localized resonances and provide more methods for wave control engineering.

### Single mode interface state

**Figure 3:**
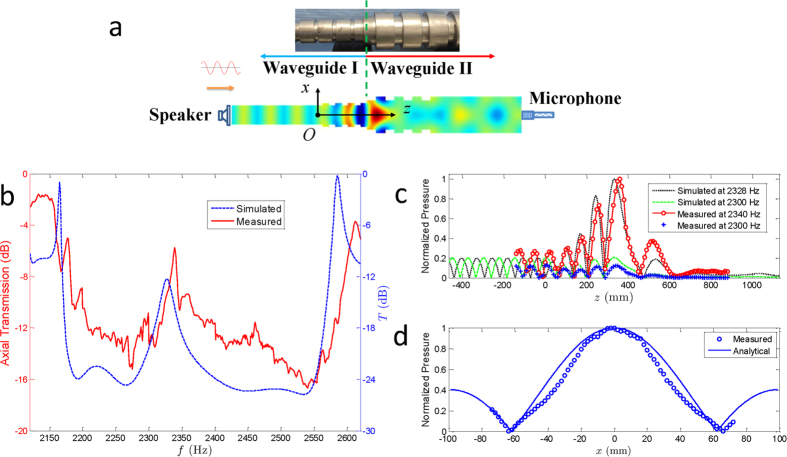
Single-mode heterostructure waveguide. (**a**) Schematic illustration of the experimental configuration. An elaborate stainless steel heterostructure waveguide is shown in the upper inset; the simulated sound pressure at 2328 Hz is also included below. The homochromatic sound waves were regulated to low-order transverse modes, propagated through the heterostructure waveguide, and detected by a microphone housed in a long tube. (**b**) Axial transmission measured in the experiments, accompanied by the energy transmission calculated by the FEM. (**c**) Normalized absolute value of sound pressure along the symmetry axes of the heterostructure waveguides. The red circles connected by the solid line and the black dotted line denote the measured and simulated results at the interface states, respectively, whereas the blue crosses and the green dashed line are the measured and simulated data away from the interface states, respectively. (**d**) Normalized absolute value of sound pressure along the *x*-axis of the single-mode interface state. The circles and the solid line depict the measured and analytical sound pressures, respectively, which have been normalized by their own maximums.

Based on the theoretical and numerical investigations, we have identified a type of acoustic heterostructure to investigate the single-mode interface states experimentally. The experimental setup is schematically illustrated in Fig. 3a; the elaborated stainless steel heterostructure waveguide is shown in the upper inset, consisting of the Bragg waveguide (Waveguide I), with Λ = 70 mm and *r*_0_ = 60 mm, and the NB (Waveguide II), with Λ = 100 mm and *r*_0_ = 90 mm. These parameters were selected to simplify fabrication and to ensure that Waveguide I and II belong, respectively, to the cyan and magenta volumes in Fig. 1f, with different topological properties (see the Supplementary Material). The simulated sound pressures at the interface state are also depicted in the waveguide schematics. The measured axial transmission in the frequency range of the band gap and interface state is illustrated in Fig. 3b, accompanied by the simulated transmission coefficient. Both the experimental and numerical results confirm the appearance of the interface state in the band gap, whose frequencies are 2340 Hz and 2328 Hz, respectively. The differences can be ascribed to slight machining imperfections. The absolute values of sound pressures, normalized to their own maximum, are shown in Fig. 3c. The interface states can effectively accumulate the sound energy at the interfaces, whereas the amplitudes are gradually attenuated along the propagation direction when the frequencies are selected away from the interface states.

Comparing the sound pressures along the symmetric axis has confirmed the appearance of the interface state in the heterostructure consisting of Waveguides I and II with different topological characteristics, but the mode structure still has not been investigated experimentally. Setting the incident sound at 2340 Hz, we carefully moved the microphone along the *z* axis to 360 mm, where the sound pressure finds its own maximum, in Fig. 3c. With the longitudinal position fixed, the microphone was moved along the *x* axis slowly, with a step of 2 mm, from −74 mm to 72 mm. The moving range is not symmetric because of bending in the long tube in which the microphone is housed. The recorded sound pressure is normalized by its maximum and depicted in Fig. 3d by the circles. We also represent the norm of the analytical Bessel function of the first mode J_0_(*k*_*r*,1_*r*/*r*_*max*_) by the solid line, where *r*_*max*_ is 99 mm. The consistency between the recorded data and the analytical curve verifies the achieved single first mode in the interface state and the effective suppression of the fundamental mode. Additionally, sound pressure was also detected along the *x* axis around the interface state (see the Supplementary Material), confirming the consistency between experiments and simulations. Connecting two acoustic waveguides with forbidden bands of different topological characteristics produced an interface state and a single first mode with very large enhanced intensity.

## Discussion

We have demonstrated that band interactions and their topological transition can be realized in a classical acoustic system. The so-called Bragg and non-Bragg gaps in a corrugated waveguide have been identified as different topologies. Continuously varying the geometry parameters of waveguides has verifiably resulted in a topological transition between the Bragg and non-Bragg gaps, in which the reflection impedance changes the sign. We have selected the geometry parameters and constructed a heterostructure waveguide with stainless steel materials. Because of the high-order-mode-induced non-Bragg gaps, the proposed heterostructures provide not only a traditional interface state but also an enhanced single high-order mode. These intriguing physical phenomena can easily be generalized to other quantum or classical systems. The substantially enhanced intensity with different mode structures could find wide application in the future in sound detection, biomedical imaging, underwater sound control, among other fields.

## Methods

### Structure preparation

We fabricated the heterostructure waveguide using stainless steel in the form of 10-mm-thick plates and 4-mm-thick pipes with different inner diameters. Waveguide I was fabricated as follows: first, we lathed the 108 mm inner diameter pipes into 35-mm-long pieces and 132 mm inner diameter pipes into 55-mm-long ones. Second, we punched the plate into flanges with an inner diameter of 116 mm and an outer diameter of 132 mm. Finally, the pieces of wide and narrow pipes were welded to the flanges. At both ends of Waveguide I, we processed the thread to simplify connection to the straight duct and Waveguide II. In a similar way, we lathed the 162 mm and 198 mm inner diameter pipes into pieces that were 50 and 70 mm long, respectively. The punched flanges had 170 mm inner and 198 mm outer diameters. After welding the wide and narrow pipes to the flanges, we processed the thread at both ends of Waveguide II. The process of manufacturing the heterostructure waveguide was completed by tightening the thread at the ends.

### Experimental configuration and measurements

Figure 3a shows the experimental configuration schematically, in which the coaxial monitor speaker is excited by a harmonic signal generated by a National Instruments card PCI-4461 and magnified by an amplifier. The excited monochromic sound radiates into a 500-mm-long straight duct with a diameter of 108 mm and is regulated into the fundamental mode before propagating into the heterostructure waveguide. Thus, we can control the homogeneity of the waves entering the heterostructure. At the other end of the structure, another straight duct is connected to reform the output impedance. A microphone (G.R.A.S. 1/4′′ free-field microphone 46BE) is housed in a 2-m-long pipe held by a 3-dimensional motorized translation stage. The translation stage can afford 3-dimensional movement of the microphone; consequently, we can detect the sound pressures throughout most of the waveguide structure. The signals received by the microphone are sampled at 200 kHz and are recorded by the PCI card. We have performed a tone-extraction analysis to identify the frequency and amplitude of the audio signal obtained.

### Numerical calculations

We performed the FEM simulations using COMSOL Multiphysics with an axisymmetric model. In simulations, the acoustic properties of air in the waveguides were set to 1.25 kg/m^3^ for the density and 343 m/s for the speed of sound. We assigned the plane-wave radiation boundary conditions to the inlet and outlet of the waveguide and the hard-boundary sound conditions to the outer boundaries. The incident and transmitted sound powers were computed using the boundary integration tool; from this information, the transmission coefficients could be calculated.

## Additional Information

**How to cite this article:** Fan, Y.-X. *et al*. Single-mode interface states in heterostructure waveguides with Bragg and non-Bragg gaps. *Sci. Rep.* **7**, 44381; doi: 10.1038/srep44381 (2017).

**Publisher's note:** Springer Nature remains neutral with regard to jurisdictional claims in published maps and institutional affiliations.

## Supplementary Material

Supplementary Information

## Figures and Tables

**Table 1:** Optimal parameters of the best fitting curves for the radius distributions of the interface states.

*f* (Hz)	*a*_0_	*a*_1_	*a*_2_
2349	0.1119	1.0650	−0.1769
2266	0.9913	−0.0033	0.0120
2562	0.1040	0.8801	0.0159
2396	0.1547	0.9516	−0.1063
