# Supplementary material for: Single-mode interface states in heterostructure waveguides with Bragg and non-Bragg gaps
Source: Sci Rep. 2017 Mar 13;7:44381. doi: 10.1038/srep44381 (PMC5347004; doi:10.1038/srep44381)
Supplement: Supplementary Information [file srep44381-s1.pdf]

Supplements:

Single-mode interface states in heterostructure waveguides with Bragg and non-Bragg gaps

Ya-Xian Fan[1, 2], Tang-Qing Sang[1], Ting Liu[1], Lan-Lan Xu[1], and Zhi-Yong Tao[1, 3]

([1] Key Lab of In-fiber Integrated Optics, Ministry Education of China, Harbin Engineering University, Harbin 150001, People's Republic of China. [2] Photonics Research Centre, College of Science, Harbin Engineering University, Harbin 150001, People's Republic of China. [3] Physics Research Centre, College of Science, Harbin Engineering University, Harbin 150001, People's Republic of China.)

1. Topological characteristics of Bragg and non-Bragg gaps:

The sound propagating in the corrugated waveguides can be formulated using the dimensionless wave equation

$$\frac{\partial^2\phi}{\partial t^2} = \nabla^2\phi \tag{S1}$$

and the hard-walled boundary condition

$$\frac{\partial\phi}{\partial r} = \varepsilon\frac{\mathrm{d}W(z)}{\mathrm{d}z}\frac{\partial\phi}{\partial z}, \tag{S2}$$

where ϕ is the velocity potential, ε is the corrugation amplitude, and $W(z)$ is a periodic function describing the wall corrugation. With the multi-scale expansions

$$z_0 = z, z_1 = \varepsilon z;\ t_0 = t, t_1 = \varepsilon t;\quad \phi = \phi_1\varepsilon + \phi_2\varepsilon^2 + \cdots, \tag{S3}$$

we obtain the first two order equations:

$$\left(\nabla_r^2 + \frac{\partial^2}{\partial z_0^2} - \frac{\partial^2}{\partial t_0^2}\right)\phi_n = F_n, \quad n = 1, 2, \tag{S4}$$

$$\frac{\partial\phi_n}{\partial r} = G_n \quad on \quad r = 1, \quad n = 1, 2, \tag{S5}$$

where

$$\begin{aligned} &F_1 = 0, \\ &F_2 = 2\frac{\partial^2\phi_1}{\partial t_0\partial t_1} - 2\frac{\partial^2\phi_1}{\partial z_0\partial z_1}, \\ &G_1 = 0, \\ &G_2 = \frac{\mathrm{d}W(z_0)}{\mathrm{d}z_0}\frac{\partial\phi_1}{\partial z_0}, \end{aligned} \tag{S6}$$

and $\nabla_r^2 = \frac{1}{r}\frac{\partial}{\partial r}\left(r\frac{\partial}{\partial r}\right)$, with the solution depending on the radius $\mathrm{J}_0\left(k_{r,m}r\right)$.

Considering the B1-NB heterostructure waveguide, we derive the coupled mode equations in the forbidden bands for each waveguide. For the Bragg gaps, the first-order solution is

$$\phi_1 = [A(z_1,t_1)\mathrm{e}^{\mathrm{i}k_z z_0} + B(z_1,t_1)\mathrm{e}^{-\mathrm{i}k_z z_0}]\mathrm{e}^{-\mathrm{i}\omega t_0} + c.c.. \tag{S7}$$

Inserting into the Green formula yields the coupled mode equations:

$$\mathrm{i}\left(\frac{\partial}{\partial z_1} + \frac{\partial}{\partial t_1}\right)A - KW_1 B = 0, \tag{S8}$$

$$\mathrm{i}\left(\frac{\partial}{\partial z_1} - \frac{\partial}{\partial t_1}\right)B + KW_{-1} A = 0, \tag{S9}$$

where the dimensionless wavenumber of the wall $K = \frac{2\pi r_0}{\Lambda}$ and $W_{\pm 1}$ is the major Fourier component of the wall function $W\left(z\right)$, which plays the major role in the wave-structure interactions. Based on the properties of waves in the gaps, we take the time dependence $\mathrm{e}^{\mathrm{i}\delta t_1}$ with the frequency detuning δ and require the sound wave to decay away from the interface with $\mathrm{e}^{\mathrm{i}\alpha z_1}$. Rearrangement and substitution lead to the relation

$$\delta^2 + \alpha^2 = K^2 \,|W_{\pm 1}|^2, \tag{S10}$$

and the reflection coefficient, in units of distance and time, is

$$R_B = -\frac{\delta + \mathrm{i}\alpha}{KW_{-1}}. \tag{S11}$$

For the non-Bragg gaps, the first-order transverse mode should be considered in addition to the fundamental one. Thus, the first-order solution should be

$$\phi_1 = [A(z_1,t_1)\mathrm{e}^{\mathrm{i}(K\text{-}k_z)z_0} + B(z_1,t_1)\,\mathrm{J}_0\left(k_{r,1}r\right)\mathrm{e}^{-\mathrm{i}k_z z_0}]\mathrm{e}^{-\mathrm{i}\omega t_0} + c.c. \tag{S12}$$

and the related couple mode equations are

$$\mathrm{i}\left(\frac{\partial}{\partial z_1} + \frac{\partial}{\partial t_1}\right)A + \mathrm{J}_0\left(k_{r,1}\right)K\kappa W_1 B = 0, \tag{S13}$$

$$\mathrm{i}\left(\kappa\frac{\partial}{\partial z_1} - \frac{\partial}{\partial t_1}\right)B - \frac{KW_{-1}}{\mathrm{J}_0\left(k_{r,1}\right)}A = 0, \tag{S14}$$

where $\kappa = \frac{K^2 - k_{r,1}^2}{K^2 + k_{r,1}^2}$. Similar derivations result in

$$\frac{(1+\kappa)^2}{4\kappa^2}\delta^2+\alpha^2=K^2\,|W_{\pm 1}|^2\,, \tag{S15}$$

and the reflection coefficient at the interface, in units of distance and time, is

$$R_{NB}=\frac{\frac{1+\kappa}{2\kappa}\delta+\mathrm{i}\alpha}{\mathrm{J}_0\left(k_{r,1}\right)KW_1}\,. \tag{S16}$$

According to the definition of relative reflection impedance, we obtain

$$\zeta=\frac{2\,\mathrm{Im}(R)}{|1-R|^2} \tag{S17}$$

in the band gaps. Thus, the sign of the relative reflection impedance is determined by the sign of the imaginary part of the reflection coefficient. Eqs. (S11) and (S16) show the different signs of impedances ζ for the Bragg and non-Bragg gaps, respectively, with respect to the different topological properties.

2. Sound pressure for heterostructure waveguides:

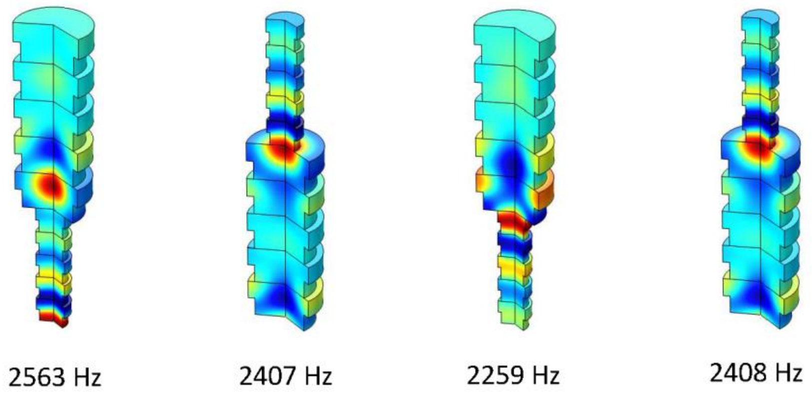

Figure S1| Sound pressure for the heterostructure waveguides connecting B2 and B3 with NB.

The sound pressure distributions for the rest of the heterostructure waveguides are illustrated in Fig. S1. The pressures for B2-NB at its second peak (2563 Hz) in the frequency range are very similar to those for B3-NB shown in Fig. 2c at 2562 Hz. Similar pressure distributions are also found for B3-NB at 2259 Hz and B2-NB at 2266 Hz (in Fig. 2c). When exchanging the connecting order, only one peak appears in each frequency range. The sound pressures for NB-B2 at 2407 Hz and NB-B3 at 2408 Hz are in accordance with those for NB-B1 at 2369 Hz presented in Fig. 2c. All of the pressure distributions indicate the interface state arising from the connection of different topological band gaps. It is clear that not all of them are single first mode interface states. When the NB is positioned first, the interface states produce a transition from the first mode to the fundamental one. For the connection of B2-NB and B3-NB, the lower frequency peaks are related to the fundamental mode interface states. Although the higher frequency peaks are responsible for the first mode interface states, the sound pressures are still very large at the inlets

of the waveguide, indicating that the energy accumulation effects are not as significant as those of the heterostructure of B1-NB.

3. Topological difference between Waveguide I and II:

In addition to the theoretical analysis of the topological properties of the Bragg and non-Bragg gaps, we have verified the π-phase shifting of the Zak phase by measuring the reflection phase of each single periodic waveguide. The sound pressures have been simulated and detected for part of the straight waveguide in which we connected B1 and NB to the measurement apparatus separately. The normalized absolute value of the sound pressure is depicted in Fig. S2. The dashed and solid lines denote the sound pressure reflected by B1 and NB, respectively; the squares and circles connected by the dotted lines are the experimental results. Both the simulated and experimental data indicate that the reflective phase difference between the Bragg and non-Bragg gaps is $\pi/2$. Considering the absolute value presented in Fig. S2, the actual difference of the Zak phase should be π, which confirms the topological difference achieved by the theoretical analysis.

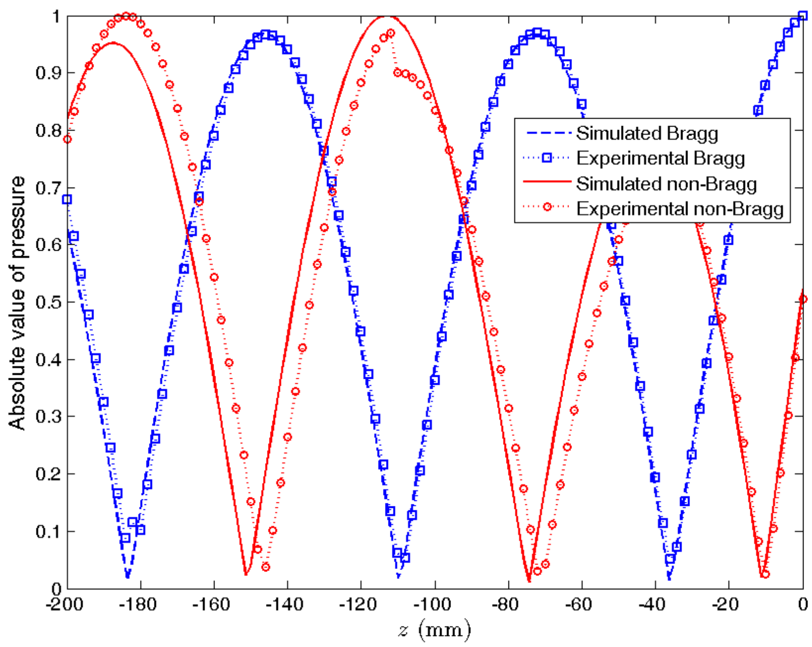

Figure S2| Measure of the Zak phase by the reflection phase, showing the topological difference between the Bragg and non-Bragg gaps.

4. Measured radius distributions:

In addition to the detected radius distributions at the maximum pressure along the longitudinal direction, as shown in Fig. 3d, we have performed measurements along the radius for eight z locations, with a step of 5 mm, around the interface state. The absolute value of measured sound pressure is normalized and depicted in Fig. S3. The results indicate a very high degree of consistency with the simulations presented in Fig. 3a. The single-mode interface state is created by the transition from the fundamental mode to the first one. It can be clearly observed that the radius distribution to the left of the longitudinal maximum is somewhat flat on top, demonstrating the effects of the fundamental mode.

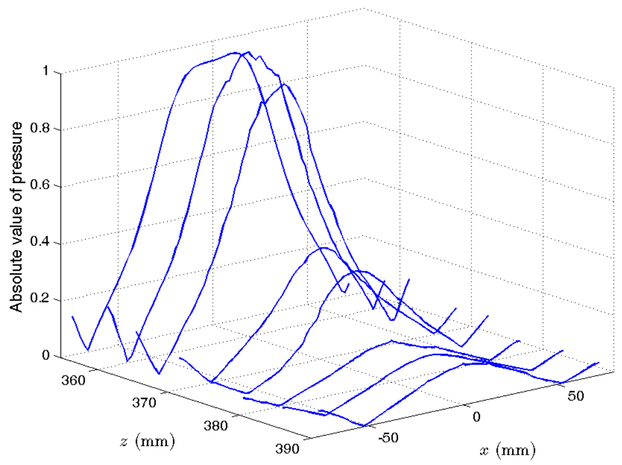

Figure S3| Measured radius distributions near the interface state, confirming consistency with the simulations.
